# Click chemistry armed enzyme-linked immunosorbent assay to measure palmitoylation by hedgehog acyltransferase

**DOI:** 10.1016/j.ab.2015.08.025

**Published:** 2015-12-01

**Authors:** Thomas Lanyon-Hogg, Naoko Masumoto, George Bodakh, Antonio D. Konitsiotis, Emmanuelle Thinon, Ursula R. Rodgers, Raymond J. Owens, Anthony I. Magee, Edward W. Tate

**Affiliations:** a Institute of Chemical Biology, Department of Chemistry, Imperial College London, South Kensington SW7 2AZ, UK; b Molecular Medicine Section, National Heart & Lung Institute, Imperial College London, South Kensington SW7 2AZ, UK; c Division of Structural Biology, Henry Wellcome Building for Genomic Medicine, University of Oxford, Roosevelt Drive, Oxford, OX3 7BN, UK; d OPPF-UK, The Research Complex at Harwell, Rutherford Appleton Laboratory, Harwell, Oxfordshire, OX11 0FA, UK

**Keywords:** Hedgehog acyltransferase, Protein palmitoylation, Click chemistry, MBOAT, PTM, posttranslational modification, HTS, high-throughput screen, GOAT, ghrelin-*O*-acyltransferase, cat–ELCCA, catalytic assay using an enzyme-linked click chemistry assay, CoA, coenzyme A, HRP, horseradish peroxidase, Hhat, hedgehog acyltransferase, MBOAT, membrane bound *O*-acyltransferase, Shh, sonic hedgehog, Hh, hedgehog, click–ELISA, click chemistry armed enzyme-linked immunosorbent assay, PCR, polymerase chain reaction, HEK293a, human embryonic kidney 293a, SDS, sodium dodecyl sulfate, PAGE, polyacrylamide gel electrophoresis, RT, room temperature, PBS, phosphate-buffered saline, YnC_15_, heptadec-16-ynoic acid, BSA, bovine serum albumin, TCEP, tris(2-carboxyethyl)phosphine, TBTA, tris[(1-benzyl-1H-1,2,3-triazol-4-yl)methyl]amine, OTG, *n*-octyl β-d-glucopyranoside, CHAPS, 3-[(3-cholamidopropyl)dimethylammonio]-1-propanesulfonate, DDM, *n*-dodecyl β-d-maltopyranoside

## Abstract

Hedgehog signaling is critical for correct embryogenesis and tissue development. However, on maturation, signaling is also found to be aberrantly activated in many cancers. Palmitoylation of the secreted signaling protein sonic hedgehog (Shh) by the enzyme hedgehog acyltransferase (Hhat) is required for functional signaling. To quantify this important posttranslational modification, many in vitro Shh palmitoylation assays employ radiolabeled fatty acids, which have limitations in terms of cost and safety. Here we present a click chemistry armed enzyme-linked immunosorbent assay (click–ELISA) for assessment of Hhat activity through acylation of biotinylated Shh peptide with an alkyne-tagged palmitoyl-CoA (coenzyme A) analogue. Click chemistry functionalization of the alkyne tag with azido-FLAG peptide allows analysis through an ELISA protocol and colorimetric readout. This assay format identified the detergent *n*-dodecyl β-d-maltopyranoside as an improved solubilizing agent for Hhat activity. Quantification of the potency of RU-SKI small molecule Hhat inhibitors by click–ELISA indicated IC_50_ values in the low- or sub-micromolar range. A stopped assay format was also employed that allows measurement of Hhat kinetic parameters where saturating substrate concentrations exceed the binding capacity of the streptavidin-coated plate. Therefore, click–ELISA represents a nonradioactive method for assessing protein palmitoylation in vitro that is readily expandable to other classes of protein lipidation.

Posttranslational modification (PTM) of proteins through S-acylation of cysteine residues with predominantly C16:0 fatty acids (termed *palmitoylation*) is one of the most common forms of PTM. The palmitoyl acyltransferase family of proteins is associated with a range of diseases, including neurological disorders and cancer [1]. Classically, protein lipidation has been studied through the application of radiolabeled lipids to facilitate detection [2]. However, such methods are limited in their utility due to requirements for lengthy detection times, the hazards of using radioactive materials, and the associated high costs of materials and disposal. With the advent of the click chemistry era, the study of protein lipidation has advanced significantly. Here, fatty acids labeled with azide or alkyne bioorthogonal reporters undergo copper(I)-catalyzed [3 + 2] cycloaddition for the attachment of reporter moieties. Such reporters have allowed detailed analysis of lipidation through a range of techniques, including Western blotting and in-gel fluorescence [3]. One of the most powerful applications of click chemistry to in vivo studies is through affinity enrichment and proteomic analysis. This approach has identified changes in the N-myristoylated proteome during the cell cycle and vertebrate development [4,5] and has been applied to validate the lipid transferase *N*-myristoyl transferase as an antimalarial drug target in *Plasmodium falciparum* [6].

During recent years, click chemistry has also been applied to in vitro studies of lipidation to afford a nonradioactive high-throughput screen (HTS) format [7]. The enzyme ghrelin-*O*-acyltransferase (GOAT), which is responsible for the octanoylation of the growth hormone releasing peptide ghrelin, was assessed through a catalytic assay using an enzyme-linked click chemistry assay (cat–ELCCA). A biotinylated substrate ghrelin peptide is bound to a streptavidin-coated plate and incubated with GOAT-containing membrane fractions and alkynyl-tagged *n*-octanoyl-CoA (coenzyme A). The resulting alkynylated peptide is then subjected to labeling via click chemistry with azido-HRP (horseradish peroxidase) to facilitate product detection through fluorogenic deacetylation of Amplex Red in the presence of hydrogen peroxide catalyzed by HRP [7]. This methodology allowed the assessment of GOAT activity and measurement of kinetic parameters along with screening to identify small molecule inhibitors [8].

Inspired by the success of the cat–ELCCA approach, we sought to develop a method to measure activity and kinetics of the enzyme hedgehog acyltransferase (Hhat). Hhat is a multipass transmembrane protein [9,10] and, like GOAT and porcupine, is a member of the membrane bound *O*-acyltransferase (MBOAT) family of enzymes. MBOATs show similar topology in regions surrounding conserved residues that are required for catalysis [11]. Hhat is responsible for the palmitoylation of sonic hedgehog (Shh), a secreted morphogen that is involved in neurogenesis during embryonic development and is aberrantly activated in mature tissues leading to carcinogenesis [12,13]. Shh is palmitoylated via an amide linkage on the N-terminal cysteine following signal peptide cleavage, most likely by initial palmitoylation on the side chain of the cysteine residue, with the palmitate group subsequently undergoing an S–N acyl shift to the N-terminus [14]. Palmitoylation of Shh has been shown to be essential for Shh signaling, thereby making Hhat an attractive target for therapeutic intervention and as a tool to investigate the hedgehog (Hh) pathway [15]. Indeed, a class of 5-acyl-6,7-dihydrothieno[3,2-*c*]pyridines was recently identified as inhibitors of Hhat using highly ionizing ^125^I-labeled palmitoyl-CoA in a scintillation proximity assay HTS [16]. However, contrary to the proposed therapeutic benefit of Hh pathway inhibition, several recent publications have demonstrated that formation of an Hh signaling promoted stromal matrix around tumors actually results in restriction of tumor growth [17–19]. This dramatic contrast in the prognosis of Hh inhibition highlights the need for better understanding of the Hh pathway, requiring both improved assays and chemical tools. To date, many studies of Hhat activity have employed radiolabeled palmitate [14,16,20–23], which may impede both the analysis and development of existing inhibitors and the identification of new alternative series with improved properties. To this end, we employed a click chemistry armed enzyme-linked immunosorbent assay (click–ELISA) format to study Hhat activity, kinetic parameters, and assessment of Hhat inhibitors.

## Materials and methods

### Plasmid construction and cell culture

Human Hhat cDNA (accession number BC117130) base pairs 4 to 1479 was amplified by polymerase chain reaction (PCR) (forward primer: AGGAGATATACCATGCTGCCCCGATGGGAACTGG; reverse primer: CAGAACTTCCAGTTTGTCCGTGGCGTAGGTCTGGGC) and inserted by ligation independent In-Fusion cloning (Takara Bio/Clontech, France) [24] into the expression vector, pOPINEneo-3C-FLAG. This vector was produced by incorporating a C-terminal 3C protease cleavage site, followed by a C-terminal FLAG and 8 × histidine epitope into the plasmid, ptriex2neo (Novagen). The human embryonic kidney 293a (HEK293a) line was transfected with pOPINEneo-Hhat-3C-FLAG-His_8_ using TurboFect (Thermo Scientific) according to the manufacturer's instructions. Single colonies were ring cloned and selected for stable transfection through culturing in Dulbecco's modified Eagle's medium (Invitrogen) supplemented with 9% fetal bovine serum (Invitrogen) and 500 μg/ml G418 (Sigma) for at least five passages. Resistant cells were assessed by anti-polyhistidine immunoblotting, and the highest expressing cell line was selected for further protein expression. Cells were passaged as described previously [15].

### Immunoblotting

Protein samples were supplemented with reducing NuPAGE Sample Buffer (Fisher), separated by sodium dodecyl sulfate–polyacrylamide gel electrophoresis (SDS–PAGE) on a 15% gel, and transferred to polyvinylidene difluoride membranes (Millipore). Membranes were blocked at room temperature (RT) for 1 h in phosphate-buffered saline (PBS) with 5% skimmed milk and then incubated with α-polyhistidine-HRP monoclonal antibody (1:3000, R&D Systems) for 16 h at 4 °C. Bound immunocomplexes were detected with ECL Plus (Pierce) and visualized on an Ettan DIGE Imager (GE Healthcare). For normalization of Hhat concentration, band intensities were quantified using ImageQuant software (GE Healthcare).

### Protein expression and solubilization

Buffers and conditions for Hhat expression and activity were taken from existing literature protocols [14] with the following modifications: HEK293a cells stably transfected with pOPINE-Hhat-3C-FLAG-His_8_ were grown to 90% confluence in 2 × T175 flasks, harvested via trypsinization, washed with 10 ml of 1 × PBS, and stored at −80 °C. Pellets were lysed as described in the literature [14]. Unlysed cells were removed by centrifugation at 1000 rcf for 10 min at 4 °C, and the resulting supernatant was separated into soluble (S100) and membrane (P100) fractions through centrifugation at 100,000 rcf for 1 h at 4 °C. The P100 fraction was resuspended in 8 ml of solubilization buffer (20 mM Hepes [pH 7.3], 350 mM NaCl, and 5% glycerol) supplemented with 1% (w/v) detergent and incubated for 60 min on ice, followed by centrifugation at 100,000 rcf for 1 h at 4 °C to afford a solubilized membrane fraction [P100(sol)] and nonsolubilized material [P100(n/sol)], which were stored at −80 °C.

### Peptide and inhibitor synthesis

Heptadec-16-ynoic acid (YnC_15_) was synthesized according to existing literature protocols [25,26] and coupled to CoA using 1,1′-carbonyl-diimidazole activation. 4-Azidobutyric acid was prepared in two steps from ethyl 4-bromobutyrate. Residues 1 to 11 of the mature Shh protein (CGPGRGFGKRK) were used as the substrate for Hhat-catalyzed reactions, and Shh(1–11)-PEG_3_-biotin, YnC_15_-Shh(1–11)-PEG_3_-biotin, and azido-FLAG (DYKDDDDK) peptides were synthesized using standard solid phase peptide synthesis protocols; full experimental information and characterization can be found in the online supplementary material. Synthesis of RU-SKI inhibitors was performed following our previously described synthetic route to access 5-acyl-6,7-dihydrothieno[3,2-*c*]pyridines [27]; full experimental information and characterization of the RU-SKI inhibitors can be found in the associated Data in Brief article [28].

### Click–ELISA

Reacti-Bind streptavidin-coated clear wells (Fisher) were activated by washing with 3 × 200 μl of wash buffer (PBS–Tween 20 + 0.1% bovine serum albumin [BSA]), followed by 3 × 200 μl of reaction buffer (100 mM MES [pH 6.5], 20 mM NaCl, 1 mM DTT [dithiothreitol], and 0.1% [w/v] BSA). YnC_15_-CoA and Shh(1–11)-biotin (in reaction buffer) were incubated with 10% (v/v) P100(sol) fraction (∼0.2 mg/ml) in a final volume of 100 μl at RT in activated streptavidin-coated wells. At the required time points, the well contents were discarded and the wells were washed with 3 × 200 μl of wash buffer, followed by 3 × 200 μl of reaction buffer. Wells were then labeled using 10 μM azido-FLAG peptide, 1 mM CuSO_4_, 1 mM tris(2-carboxyethyl)phosphine (TCEP), and 1 mM tris[(1-benzyl-1H-1,2,3-triazol-4-yl)methyl]amine (TBTA) in wash buffer for 1 h at RT and subsequently washed with 3 × 200 μl of wash buffer and 3 × 200 μl of reaction buffer. Afterward, wells were probed with α-FLAG-HRP (1:20,000) (Sigma) in wash buffer for 1 h at RT and then washed with 3 × 200 μl of wash buffer, followed by 3 × 200 μl of reaction buffer. α-FLAG-HRP (Sigma) was visualized using BD OptEIA TMB reagent (Becton Dickinson) according to the manufacturer's protocol. The development reaction was stopped by the addition of 50 μl 1 M H_2_SO_4_. Wells were read for optical density at 450 nm on a BioTek PowerWave XS Microplate Spectrophotometer.

IC_50_ values for RU-SKI inhibitors were measured over a six-log unit serial dilution from 100 μM, corrected for background against heat-inactivated P100(sol) low control, and normalized to DMSO (dimethyl sulfoxide) vehicle-only high control.

For stopped assay conditions, reaction mixes were prepared as stated previously in 1.5-ml microfuge tubes. At the required time points, the Hhat-catalyzed reaction was stopped by the addition of 10 μl of 10% (w/v) SDS. The reaction mixtures were transferred to activated streptavidin-coated wells and incubated for 45 min at RT. Well contents were then discarded, wells were washed as described previously, and the level of Shh(1–11)-acylation was assessed via click–ELISA. Kinetic parameters were measured at saturating concentrations of 5.0 μM YnC_15_-CoA and 3.0 μM Shh(1–11) peptide and were corrected for background signal from heat-inactivated P100(sol).

## Results and discussion

### Increase in solubilized Hhat activity due to *n*-dodecyl β-d-maltopyranoside

**Fig.1:**
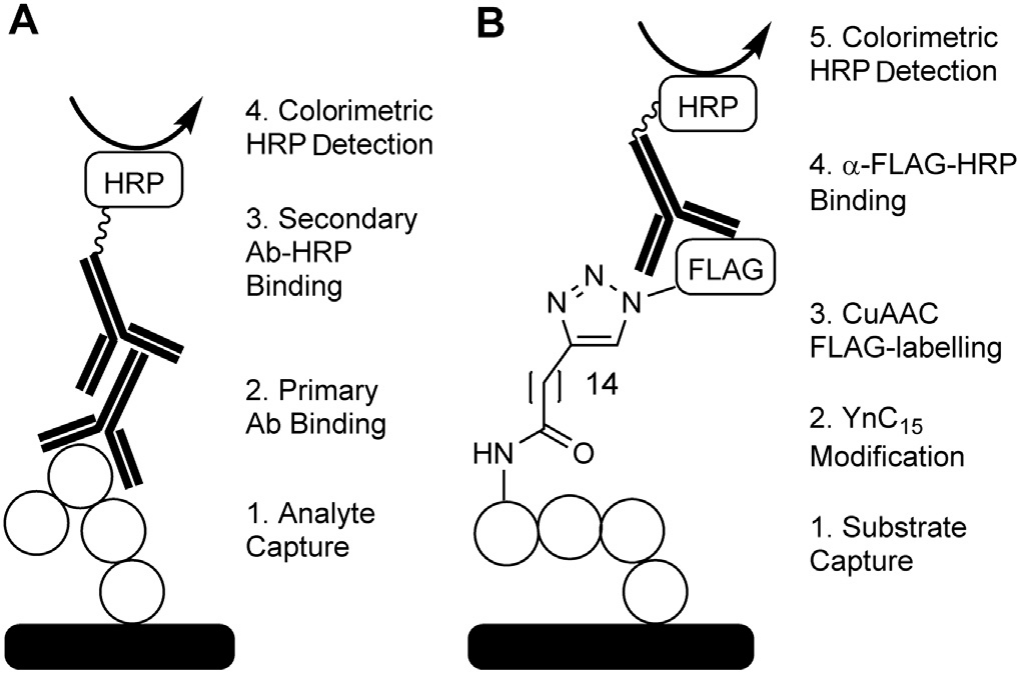
Comparison of enzyme-linked immunosorbent assay (ELISA) (A) and click chemistry armed ELISA (click–ELISA) (B). (A) Analyte is captured on the plate and detected with primary antibody, followed by secondary antibody-HRP conjugate. Colorimetric readout is proportional to bound analyte. (B) Biotinylated substrate modified with YnC_15_ alkyne tag is captured on a streptavidin-coated plate and functionalized with FLAG-tag via copper(I)-catalyzed azide-alkyne cycloaddition (CuAAC). FLAG-tag is detected by α-FLAG-HRP conjugate. Colorimetric readout is proportional to alkyne-tagged substrate.

ELISAs employ an immobilized antigen that is probed with an antibody, and subsequently an antibody-HRP conjugate, to allow the determination of levels of bound antigen through colorimetric readout (Fig. 1A). By analogy, the click–ELISA format employed an immobilized Shh(1–11) substrate peptide that was acylated by Hhat using the alkynyl-tagged palmitoyl-CoA analogue YnC_15_-CoA. The alkynylated peptide was then subjected to copper(I)-catalyzed click reaction functionalization with azido-FLAG peptide to ligate a FLAG tag. Subsequent probing with α-FLAG-HRP allowed determination of the level of Hhat-catalyzed Shh(1–11) labeling through colorimetric readout using the TMB reaction (Fig. 1B).

**Fig.2:**
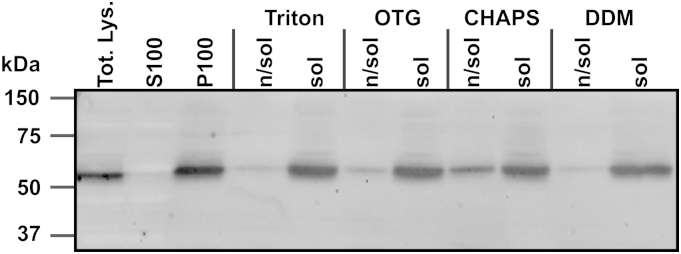
Solubilization of P100 membrane fractions. Total lysate (Tot. Lys.) was separated into S100 and P100 fractions at 100,000 rcf for 1 h. P100 membranes were solubilized in 1% (w/v) detergent for 1 h and then separated into P100(sol) and P100(n/sol) fractions at 100,000 rcf for 1 h. Samples were separated by SDS–PAGE and analyzed by immunoblotting with α-polyhistidine-HRP. *n*-octyl β-d-gluc*o*pyranoside (OTG), Triton X-100 (Triton), 3-[(3-cholamidopropyl)dimethylammonio]-1-propanesulfonate (CHAPS), and *n*-dodecyl β-d-maltopyranoside (DDM). Blot representative of three separate experiments.

Literature for the isolation of active Hhat fractions indicates that *n*-octyl β-d-glucopyranoside (OTG) affords maximal Shh palmitoylation activity [14]; however, our initial experiments following these protocols did not afford a high level of Shh(1–11) acylation (data not shown). To increase the palmitoylation activity of Hhat, alternative detergents were investigated for their ability to fully solubilize Hhat in P100 membranes while maintaining enzymatic activity. Isolated P100 membrane fractions from HEK293a cells stably expressing Hhat-FLAG-His_8_ were resuspended in solubilization buffer containing either 1% (w/v) Triton X-100, OTG, 3-[(3-cholamidopropyl)dimethylammonio]-1-propanesulfonate (CHAPS), or *n*-dodecyl β-d-maltopyranoside (DDM) and incubated for 1 h on ice. The extent of Hhat solubilization was compared by α-His immunoblotting of solubilized [P100(sol)] and nonsolubilized [P100(n/sol)] material (Fig. 2). Hhat was detected in the P100 fraction and absent in the S100 fraction. All detergents afforded high levels of solubilization, with only CHAPS showing appreciable retention of Hhat in the P100(n/sol) fraction.

The corresponding acylation activities of the detergent-solubilized P100(sol) fractions were measured using the click–ELISA format (Table 1). Moderate levels of acylation were observed from nonsolubilized P100 membranes or from OTG P100(sol) fractions. However, 2- and 5-fold increases in Shh(1–11) palmitoylation were observed from CHAPS and DDM P100(sol) fractions, respectively. No palmitoylation of Shh(1–11) peptide was observed from the S100 fraction, DDM solubilization buffer only, or the Triton X-100 P100(sol). These findings are consistent with the absence of Hhat in these fractions or the denaturing nature of Triton X-100. Subsequent experiments, therefore, employed DDM-solubilized P100(sol) fractions as a crude source of Hhat.

### Development of an in vitro click–ELISA to measure Shh(1–11) palmitoylation by Hhat

**Fig.3:**
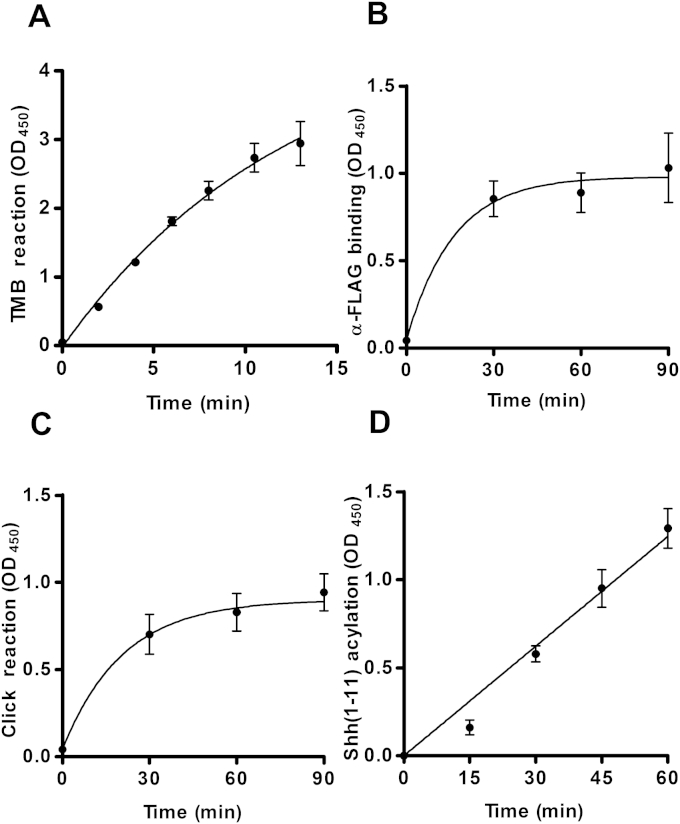
Determination of click–ELISA parameters. All assays were performed at a total Shh(1–11) peptide concentration of 1 μM. Alkynylated Shh(1–11) peptides were click chemistry functionalized with 10 μM azido-FLAG, 1 mM CuSO_4_, 1 mM TCEP, and 1 mM TBTA for 1 h unless otherwise stated. FLAG-labeled Shh(1–11) was detected with α-FLAG-HRP at 1:20,000 dilution for 1 h unless otherwise stated. TMB reactions were developed for 10 min unless otherwise stated. (A) TMB development reaction time course of YnC_15_-Shh(1–11) (0.4 μM) in the presence of Shh(1–11) (0.6 μM). Assays were performed in duplicate, *n* = 3. (B) α-FLAG-HRP binding time course of YnC_15_-Shh(1–11) (0.1 μM) in the presence of Shh(1–11) (0.9 μM) (*n* = 3). (C) Click reaction time course of YnC_15_-Shh(1–11) (0.1 μM) in the presence of Shh(1–11) (0.9 μM) (*n* = 3). (D) P100(sol)-catalyzed acylation of Shh(1–11) (1 μM) in the presence of YnC_15_-CoA (1 μM). Assays were performed in duplicate, *n* = 3.

Having identified conditions that increased Hhat acylation activity, assay parameters were validated to ensure that they were within required ranges by using the synthetic product peptide, YnC_15_-Shh(1–11)-PEG_3_-biotin. YnC_15_-Shh(1–11) peptide (0.4 μM) was processed via click–ELISA, and the TMB development reaction was measured as a function of time, indicating linearity to OD_450_ ∼ 2.00 (Fig. 3A). Binding of α-FLAG-HRP to 0.1 μM FLAG functionalized YnC_15_-Shh(1–11) product peptide was investigated as a function of time by discarding well contents at required time points and was found to be complete within 1 h of incubation (Fig. 3B). Likewise, click chemistry functionalization of 0.1 μM YnC_15_-Shh(1–11) peptide with azido-FLAG was complete within 1 h (Fig. 3C).

Prior to use in enzymatic reactions, Hhat levels in DDM-solubilized P100(sol) fractions were normalized by α-polyhistidine immunoblotting and densitometry measurement to account for variation in expression and solubilization. The extent of Shh(1–11) acylation catalyzed by approximately 2 μg P100(sol) was measured against time, which indicated the rate of palmitoylation to be essentially linear over 30 min (Fig. 3D).

### Assessment of small molecule inhibition of Hhat

**Fig.4:**
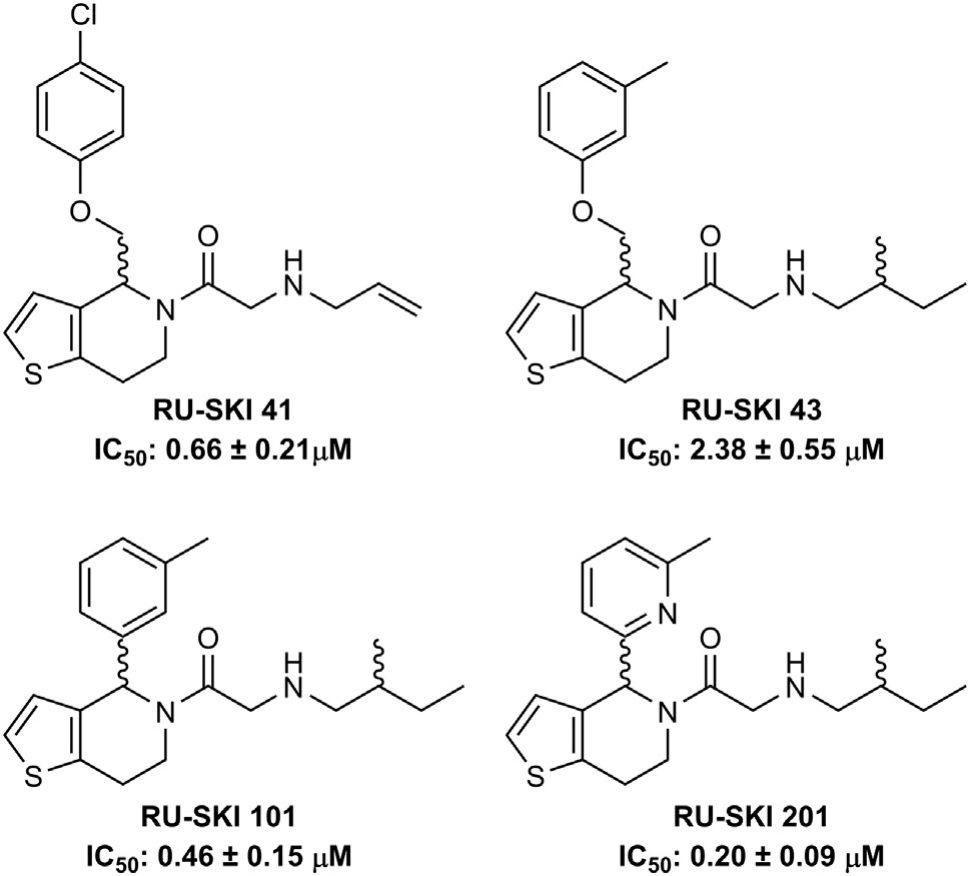
Structures of RU-SKI inhibitors and activity as assessed by click–ELISA. Inhibitors were synthesized as described in the supplementary material and were assayed over a six-log unit serial dilution from 100 μM. Samples were background corrected against heat-inactivated P100(sol) low control and normalized to vehicle-only high control. Assays were performed in duplicate, *n* = 3.

To assess the suitability of the click–ELISA for inhibition studies, the most active Hhat inhibitors described in the literature were tested for their effect on Shh(1–11) acylation [16,29]. The RU-SKI class of inhibitors are based on a 5-acyl-6,7-dihydrothieno[3,2-*c*]pyridine core (Fig. 4) and were synthesized via our previously reported synthetic strategy [27]. Full synthetic details of the RU-SKI inhibitors are provided in the associated Data in Brief article [28]. RU-SKI 41, 43, 101, and 201 were assayed over a six-log unit serial dilution from 100 μM, with all inhibitors achieving complete inhibition of Hhat at 100 μM (see Fig. S1 in supplementary material). IC_50_ values were calculated to be in the low- or sub-micromolar range (Fig. 4). This finding is in agreement with literature data for these compounds [29]. RU-SKI 201 displayed the highest potency, and RU-SKI 43 displayed the lowest potency, for Hhat inhibition of the compounds tested.

### Determination of kinetic parameters for Shh(1–11) acylation

Literature indicates that Hhat possesses lower affinity for its palmitoyl-CoA and Shh substrates than GOAT for its corresponding octanoyl-CoA and ghrelin substrates. GOAT, as assessed by cat–ELCCA, exhibits a *K*_m_ of 67.5 nM for Oct-CoA and a *K*_m_ of 99.8 nM for ghrelin [7]. Hhat has a reported *K*_m_ of 1.25 μM for palmitoyl-CoA and a reported *K*_m_ of 3.0 μM for Shh [14]. To determine apparent *K*_m_ values in a two-substrate system, one substrate must be at saturating concentration while the reaction rate in response to the second substrate concentration is assessed. Therefore, determining kinetic parameters for Hhat necessitated measuring enzyme activity at Shh(1–11) concentrations above the biotin-binding capacity of currently available streptavidin-coated plates (1.25 μM).

The assay format, therefore, was modified to measure Hhat-catalyzed palmitoylation of Shh(1–11) above plate-saturating concentrations. Hhat-catalyzed reactions were performed in microfuge tubes followed by subsequent capturing of a representative proportion of the reaction mixture on the streptavidin-coated plate for analysis. To stop Hhat-catalyzed lipidation prior to plate binding, SDS was added to a final concentration of 0.9% (w/v), which would halt the Hhat-catalyzed reaction while not disrupting the biotin–streptavidin interaction.

**Fig.5:**
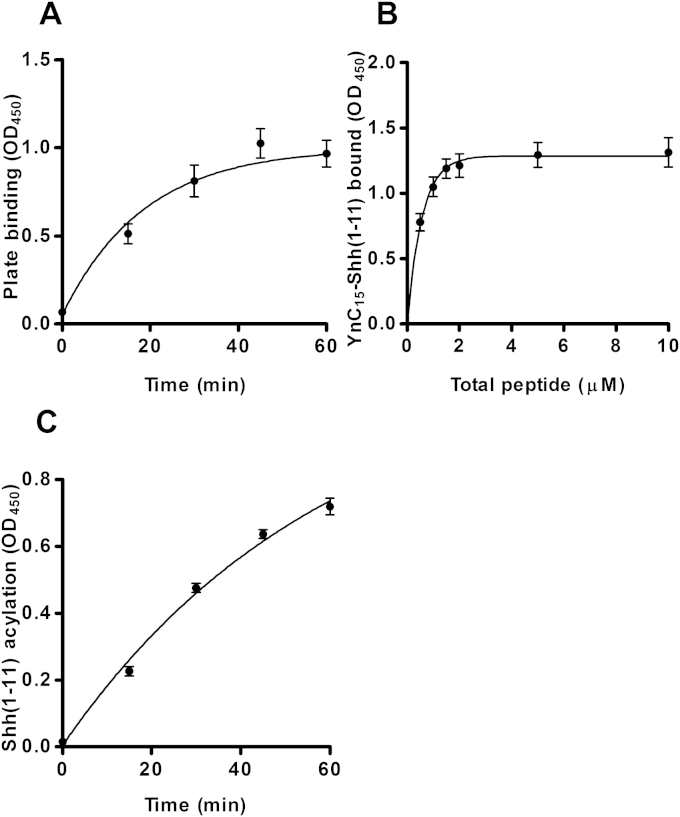
Validation of SDS stop conditions for click–ELISA kinetic analysis. The click–ELISA protocol was performed as described in Fig. 3. (A) Plate binding time course for YnC_15_-Shh(1–11) (0.1 μM) and Shh(1–11) (0.9 μM) in the presence of 0.9% SDS. Assays were performed in duplicate, *n* = 3. (B) Click–ELISA signal in the presence of varying total peptide concentrations of 40% YnC_15_-Shh(1–11)/Shh(1–11) mixture in the presence of 0.9% SDS. Assays were performed in duplicate, *n* = 3. (C) P100(sol)-catalyzed acylation of Shh(1–11) (1 μM) in the presence of YnC_15_-CoA (1 μM) stopped by the addition of SDS to 0.9% (10%, w/v, 10 μl). Assays were performed in duplicate, *n* = 3.

Binding of the YnC_15_-Shh(1–11) product peptide to the streptavidin-coated plate in the presence of 0.9% SDS was allowed to proceed for specified time periods, and then well contents were discarded and wells were analyzed by click–ELISA. The corresponding data indicated that plate binding in the presence of 0.9% SDS was essentially completed within 45 min (Fig. 5A). To determine whether both substrate and product biotinylated peptides had equal affinity for streptavidin-coated wells, and therefore would allow capture of a representative proportion of the reaction mixture, the synthetic product and substrate peptides were prepared in a 1.0:1.5 molar ratio, respectively. Total peptide concentrations ranging from 0.5 to 10 μM were prepared in the presence of 0.9% SDS, added to streptavidin-coated wells, and allowed to bind for 45 min prior to analysis. Above a total peptide concentration of 1.25 μM, the stated plate binding capacity, the OD_450_ signal remained constant, which indicated a proportional capture of the product–substrate mixture (Fig. 5B). It should be noted that the peptide sequence affected the streptavidin capture rate because alkynyl-tagged biotinylated peptides with different sequences did not afford representative capture of mixtures with Shh(1–11) substrate peptide (data not shown). The ability of 0.9% SDS to halt Hhat-catalyzed acylation was tested by the addition of the detergent to enzyme reactions at required time points, followed by incubation in streptavidin-coated wells for 45 min and analyzing wells as described previously. The data indicated a linear reaction rate over 30 min, as demonstrated previously (Fig. 5C).

Kinetic analysis of the Hhat-containing P100(sol) fractions was performed by using the SDS stop conditions and proportional capture of the reaction mixture. The level of YnC_15_-Shh(1–11) formed in the Hhat-catalyzed reaction was calibrated against a standard curve generated from serial dilution of synthetic YnC_15_-Shh(1–11) peptide. At 3.0 μM Shh(1–11) peptide, YnC_15_-CoA was found to have a *V*_max_ of 0.87 ± 0.06 pmol/min and an apparent *K*_m_ of 0.15 ± 0.05 μM (see Fig. S2A in supplementary material). At 5.0 μM YnC_15_-CoA, the Shh(1–11) peptide was found to have a *V*_max_ of 1.08 ± 0.06 pmol/min and an apparent *K*_m_ of 1.37 ± 0.25 μM (Table 2 and Fig. S2B).

## Conclusions

Click chemistry-based methods have greatly advanced understanding of Shh lipidation [9,30,31]. In the current study, we have developed a click chemistry-based ELISA format for measuring Hhat-catalyzed acylation of Shh (residues 1–11) using an alkyne-tagged palmitoyl-CoA substrate. Alkynylated product peptides were functionalized via click chemistry with azido-FLAG and then probed with α-FLAG-HRP to allow colorimetric readout of Shh(1–11) palmitoylation. Detergent screening using this assay format identified DDM as a superior solubilizing agent to OTG for Hhat activity, affording a 5-fold increase in acylation activity (Table 1), due to the milder disruptive effect on membranes of DDM compared with OTG [32]. Therefore, DDM should be adopted for future studies of solubilized Hhat activity. The click–ELISA format was successfully applied to quantify Hhat inhibition by small molecules and has also been employed to investigate the activity of various Hhat point mutants [9]. Furthermore, the assay protocol was developed to allow the screening of biotinylated substrate concentrations above the assay plate binding capacity by using stopped reaction conditions and proportional sampling of the substrate–product mixture.

The apparent *K*_m_ for Shh(1–11) of DDM-solubilized P100(sol) fractions was in agreement with literature values of OTG-solubilized Hhat (1.37 and 1.25 μM, respectively). In contrast, the apparent *K*_m_ for YnC_15_-CoA of DDM-solubilized P100(sol) fractions was lower than literature values for the apparent *K*_m_ for [^125^I]iodopalmitoyl-CoA of OTG-solubilized Hhat (0.15 and 3.0 μM, respectively) (Table 2 and Fig. S2) [14]. The stopped reaction protocol renders the assay applicable to kinetic assessment of systems with higher *K*_m_ values and, thus, expands the repertoire of enzymes that may be assayed by click chemistry–ELISA formats. The use of stopped assay conditions allows assessment of enzyme kinetics in solution phase, which avoids issues associated with using solid supported substrates (e.g., substrates bound to streptavidin-coated plates or beads). Solid supported substrates can interfere with enzyme kinetics because the substrate is not free to diffuse in solution, resulting in areas of increased local substrate concentration. In addition, the presence of the solid support itself may serve to attract or repel the enzyme, or hinder access to the substrate, thereby altering reaction rates. In solution phase studies, there is an even concentration of free substrate and the enzyme can be assumed to follow Michaelis–Menten kinetics. SDS stop conditions provide an effective means to access solution phase kinetic studies because streptavidin is unusual among proteins in its ability to tolerate high SDS concentrations while maintaining an active conformation.

Because the presented assay format employs alkynylated rather than radiolabeled lipids, it offers significant advantages in terms of safety, cost, and synthetic accessibility of substrate compounds for future lipidation investigations. The click chemistry armed enzyme-linked immunosorbent assay format, therefore, represents a powerful and versatile tool for in vitro studies of lipidation of peptide substrates by MBOAT enzymes.

## Figures and Tables

**Table 1:** Acylation activity of lysate fractions and P100(sol) fractions solubilized in the presence of different detergents.

Fraction	OD_450_	Relative activity (%)
P100	0.24 ± 0.03	27 ± 1
S100	0.05 ± 0.00	−2 ± 1
P100(Triton)	0.07 ± 0.01	1 ± 1
P100(OTG)	0.21 ± 0.07	18 ± 7
P100(CHAPS)	0.37 ± 0.09	41 ± 6
P100(DDM)	0.76 ± 0.14	100
DDM buffer	0.06 ± 0.01	0

Relative activity is background corrected to DDM buffer signal and reported as percentage relative to P100(DDM) signal. Detergent abbreviations are as described in Fig. 2. Values represent means ± standard errors. Assays were performed in duplicate, *n* = 2.

**Table 2:** *V*_max_ and apparent *K*_m_ values for YnC_15_-CoA and Shh(1–11) substrates assessed by click–ELISA at 3.0 μM Shh(1–11) and 5.0 μM YnC_15_-CoA, respectively.

Substrate	*V*_max_ (pmol/min)	Apparent *K*_m_ (μM)
Shh(1–11)	1.08 ± 0.06	1.37 ± 0.25
YnC_15_-CoA	0.87 ± 0.06	0.15 ± 0.05

Values represent means ± standard errors. Assays were performed in duplicate: Shh(1–11), *n* = 3; YnC_15_-CoA, *n* = 4.
