# Supplementary material for: Click chemistry armed enzyme-linked immunosorbent assay to measure palmitoylation by hedgehog acyltransferase
Source: Anal Biochem. 2015 Dec 1;490:66–72. doi: 10.1016/j.ab.2015.08.025 (PMC4615133; doi:10.1016/j.ab.2015.08.025)
Supplement: Supplementary file 1 [file mmc1.pdf]

Click-Chemistry Armed Enzyme Linked Immunosorbent Assay to Measure Palmitoylation by Hedgehog Acyltransferase

Thomas Lanyon-Hogg[a], Naoko Masumoto[a], George Bodakh[a], Antonio D. Konitsiotis[b,1], Emmanuelle Thinon[a,2], Ursula R. Rodgers[b], Raymond J. Owens[c], Anthony I. Magee[b,*] and Edward W. Tate[a,*]

[a] Department of Chemistry, Imperial College London, SW7 2AZ, UK; Institute of Chemical Biology, Imperial College London, SW72AZ, UK. Tel: +44 (0)20 7594 3752. E-mail: e.tate@imperial.ac.uk

[b] Molecular Medicine Section, National Heart & Lung Institute, Imperial College London, SW7 2AZ, UK. Tel: +44 (0)20 7594 3135. E-mail: t.magee@imperial.ac.uk

[c] Division of Structural Biology, Henry Wellcome Building for Genomic Medicine, University of Oxford, Roosevelt Drive, Oxford, OX3 7BN, UK & OPPF-UK, The Research Complex at Harwell, Rutherford Appleton Laboratory, Harwell, Oxfordshire, OX11 0FA, UK.

[1] Present address: Max Planck Institute of Molecular Physiology, Department of Systemic Cell Biology, Otto-Hahn-Str. 11, 44227 Dortmund, Germany.

[2] Present address: The Rockefeller University, 1230 York Avenue, New York, New York, USA

* Corresponding authors

Subject Category

Enzymatic Assays and Analyses

Contents

1. Supplemental Figures 3

2. YnC_{15} Synthesis 5

3. 4-azidobutyric acid Synthesis 9

4. Peptide Synthesis 11

5. References 14

1. Supplemental Figures

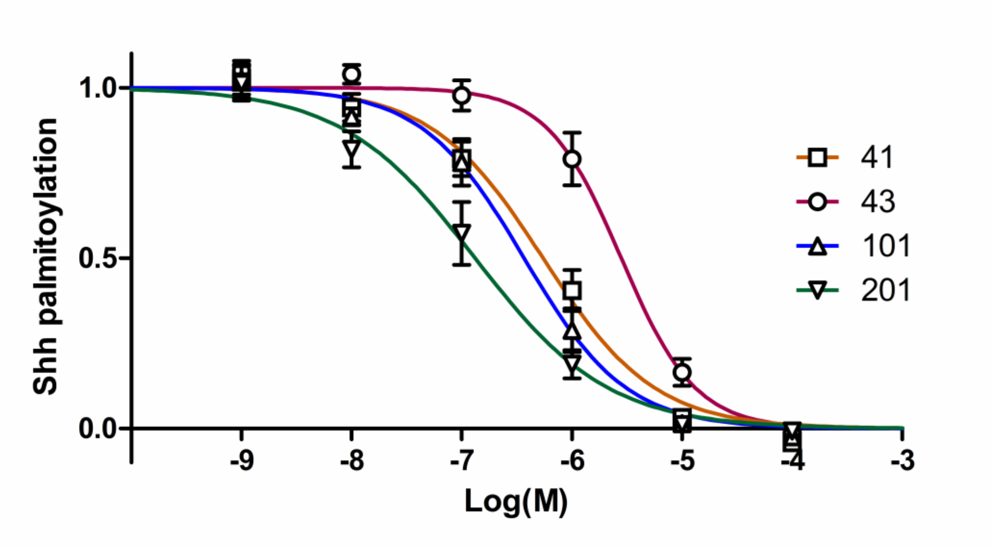

Figure S1. Dose-response curves for RU-SKI inhibition of P100(sol)-mediated YnC_{15} labelling of Shh(1-11) peptide in the presence of 1 μM YnC_{15}-CoA and 1 μM Shh(1-11). Values represent mean ± SEM (assays performed in duplicate, n = 3).

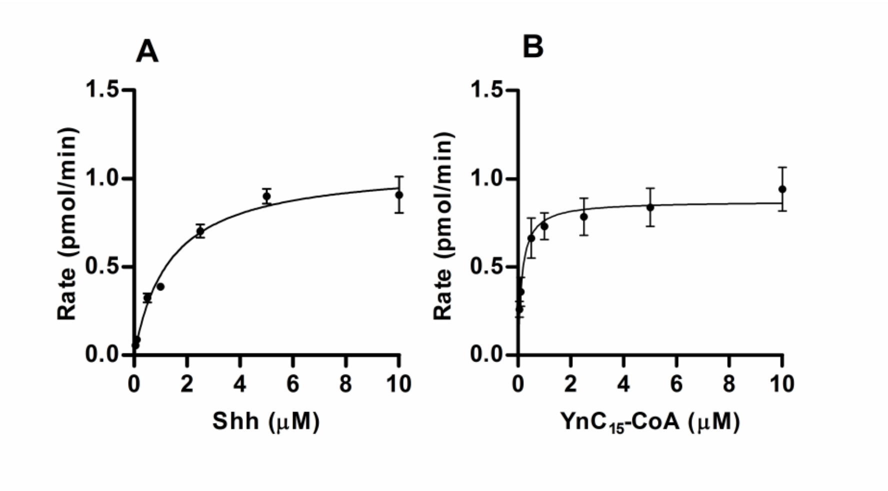

Figure S2. Kinetic analysis of P100(sol)-mediated YnC_{15} labelling of Shh(1-11) peptide using 0.9 % SDS stop and proportional capture protocol. A) Shh(1-11) kinetic parameters recorded at 5.0 µM YnC_{15}-CoA. B) YnC_{15}-CoA kinetic parameters recorded at 3.0 µM Shh(1-11). Values represent mean ± SEM (assays performed in duplicate, Shh(1-11) n = 3, YnC_{15}-CoA n = 4).

2. Materials

All reagents were purchased from commercial sources (Sigma-Aldrich, Fisher Scientific, Acros Organics) and were used without further purification. Microwave reactions were performed using a Biotage Initiator. ^{1}H NMR and ^{13}C NMR spectra were recorded at room temperature either at 400 MHz and 100 MHz on a Bruker AM-400, or at 500 MHz or 125 MHz on a Bruker AM-500 instrument. Chemical shifts (δ) are reported in parts per million (ppm) relative to residual solvent peaks as internal standard. Coupling constants (*J*) are reported in Hertz (Hz). COSY and HSQC spectra were recorded at 400 MHz (Bruker AM-400) and 500 MHz (Bruker AM-500) at room temperature. High resolution mass spectrometry (HRMS) was performed using electrospray ionisation (ESI) on an AUTOSPEC P673 spectrometer.

3. Abbreviations

EDC (1-Ethyl-3-(3-dimethylaminopropyl)carbodiimide), PyBOP ((Benzotriazol-1-yloxy)tripyrrolidinophosphonium hexafluorophosphate), TEA (Triethylamine), DIPEA (N,N-Diisopropylethylamine), DMF (Dimethylformamide), DCM (Dichloromethane), HOBt (Hydroxybenzotriazole), TFA (Trifluoroacetic acid), HBTU (*N,N,N′,N′*-Tetramethyl-*O*-(1*H*-benzotriazol-1-yl)uronium hexafluorophosphate), NMM (*N*-Methylmorpholine), TIPS (Triisopropylsilane), DTT (dithiothreitol), NMP (*N*-Methyl-2-pyrrolidone), chex (cyclohexane).

4. YnC_{15} Synthesis

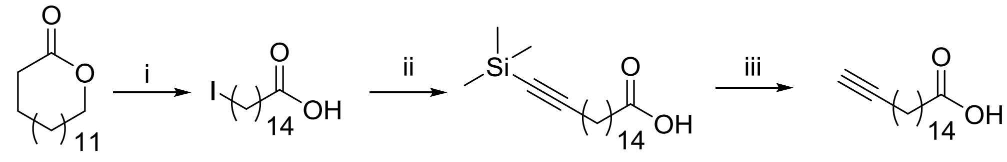

1 **2** **3**

Reagents and conditions: i) HI, AcOH, reflux ii) TMS-acetylene, *n*-BuLi (2.5 M in hexanes), DMPU, dry THF, -78 °C then room temperature iii) K_2CO_3, MeOH, room temperature.

15-Iodopentadecanoic acid (1)

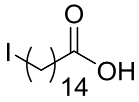

Pentadecanoline (2.2 mL, 8.3 mmol, 1 eq) was added to a mixture of hydrogen iodide (57 % in H_2O, 8 mL) and AcOH (4 mL). The reaction mixture was heated under reflux for 3 h, then allowed to cool to room temperature. A brown precipitate was obtained. The reaction mixture was quenched with a saturated solution of Na_2SO_7 (50 mL) and DCM (50 mL) was added to dissolve the precipitate. The layers were separated and the aqueous layer extracted with DCM. The combined organic layers were washed with brine, dried over Na_2SO_4, filtered and concentrated *in vacuo*. The yellow residue was purified by flash chromatography (gradient chex:EtOAc:AcOH 94:5:1 to 74:25:1) to yield **1** as white crystals (2.28 mg, 75%). Rf (chex:EtOAc:AcOH 8:2:0.1) = 0.22; ^{1}H NMR (400 MHz, $CDCl_3$) δ 3.17 (t, J = 7.1, 2H), 2.33 (t, J = 7.5, 2H), 1.80 (dt, J = 14.5, 7.1, 2H), 1.66 – 1.56 (m, 2H), 1.41 – 1.21 (m, 20H); ^{13}C NMR (101 MHz, $CDCl_3$) δ 179.42, 34.10, 33.79, 30.73, 29.80, 29.75, 29.63, 29.45, 29.27, 28.76, 24.89; HRMS (ESI, negative mode) found

413.1188 ([M – H + HCOO$^-$]$^-$ requires 413.1189). The analysis was consistent with the NMR and MS analysis reported in the literature.[1]

TMS-protected Heptadec-16-ynoic acid (2)

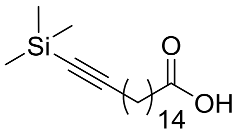

A solution of TMS-acetylene (920 µL, 2.58 mmol, 2.5 eq) in dry THF (6 mL) was cooled to -78 °C under nitrogen atmosphere using an acetone-dry ice bath. A solution of *n*-BuLi in hexanes (3.1 mL, 2.5 M solution, 3.0 eq) was added dropwise. The clear reaction mixture was allowed to warm to room temperature for 10 minutes and then cooled to -78 °C. DMPU (6.5 mL, 54 mmol, 21 eq) and a solution of 15-iodopentadecanoic acid (**1**) (950 mg, 2.58 mmol, 1.0 eq) in dry THF (6 mL) was added dropwise. The yellow reaction mixture was allowed to warm to room temperature and stirred overnight. The reaction mixture was cooled to -78 °C and quenched by dropwise addition of saturated NH_4Cl (20 mL). Et_2O (50 mL) and water (20 mL) were added, the layers separated and the aqueous layer extracted with Et_2O (2 x 50 mL). The combined organic layers were washed with water (3 x 20 mL), brine (2 x 20 mL), dried over Na_2SO_4, filtered and concentrated *in vacuo*. The brown residue was purified by flash chromatography (gradient chex:EtOAc:AcOH 99:0:1 to 74:25:1) to yield **2** as a white solid (320 mg, 37%). Rf (chex:EtOAc:AcOH 8:2:0.1) = 0.3; ^{1}H NMR (400 MHz, $CDCl_3$) δ 2.33 (t, J = 7.5, 2H), 2.19 (t, J = 7.2, 2H), 1.61 (dt, J = 14.9, 7.4, 2H), 1.49 (m, 2H), 1.27 (d, J = 28.4, 20H), 0.13 (m, 9H); HRMS (ESI, negative mode) found 265.2156 ([M – H –TMS) requires 265.2173

Heptadec-16-ynoic acid (3)

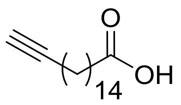

To a suspension of TMS-protected alkyne (**2**) (310 mg, 1 eq, 0.92 mmol) in MeOH (15 mL) was added K_2CO_3 (253 mg, 1.83 mmol, 2 eq). The reaction mixture was stirred at room temperature overnight. The mixture was concentrated *in vacuo*, and the residue taken up in 2 M HCl (20 mL) and Et_2O (30 mL). The layers were separated and the aqueous layer extracted with Et_2O (2 x 30 mL). The combined organic layers were washed with water (20 mL), brine (20 mL), dried over Na_2SO_4, filtered and concentrated *in vacuo* to yield **3** as an off-white solid (230 mg, 94%). The product did not require further purification. ^1H NMR (400 MHz, $CDCl_3$) δ 2.33 (t, J = 7.5, 2H), 2.16 (td, J = 7.1, 2.6, 2H), 1.92 (t, J = 2.6, 1H), 1.60 (dd, J = 7.3, 14.8, 2H), 1.55 – 1.45 (m, 2H), 1.30 (m, 20H); ^{13}C NMR (101 MHz, $CDCl_3$) δ 178.90, 84.86, 68.04, 33.84, 29.63, 29.52, 29.44, 29.25, 29.13, 29.07, 28.79, 28.52, 24.70, 18.42; HRMS (ESI, negative mode) found 265.2170 ($[M - H]^-$ requires 265.2168). The analysis was consistent with the NMR and MS analysis reported in the literature.[2]

YnC_{15}-CoA

To a suspension of pentadec-15-ynoic acid (**3**) (16.6 mg, 65 μmol, 2 eq) in dry THF (1.0 mL) was added a solution of 1,1'-carbonyl-diimidazole (12.7 mg, 78 μmol, 2.4 eq) in DCM (1.0 mL) under an atmosphere of nitrogen and stirred for 45 minutes at room temperature. The reaction mixture was concentrated *in vacuo*, and the residue dissolved in dry THF (1.0 mL). Coenzyme A hydrate from yeast (25 mg, 32.6

μmol, 1 eq) was dissolved in an aqueous solution of $NaHCO_3$ (0.5 M, 3.4 mL) and added to the solution of activated acid, and stirred at room temperature for 3 h under nitrogen. THF was removed *in vacuo*, and the product precipitated by adding 20% perchloric acid (1 mL) dropwise. The white solid was pelleted by centrifugation, washed with 1 % perchloric acid, and the precipitate dessicated overnight. The product was purified by preparative RP-HPLC over a gradient of 25 mM ammonium bicarbonate pH 8 and MeOH: 0 min 50% MeOH, 0-10 min up to 98% MeOH, 10-15 min 98% MeOH, 15-16 min 98 to 5% MeOH, 16-20 min 5% MeOH. YnC_{15}-CoA was obtained as a white lyophilised solid (9.5 mg, 29% yield). LC-MS**:** R_T=12.54 min (Absorption wavelength = 258 nm), m/z (positive mode) = 1016.34, m/z (negative mode) = 1014.23, Purity > 95%.

5. 4-azidobutyric acid Synthesis

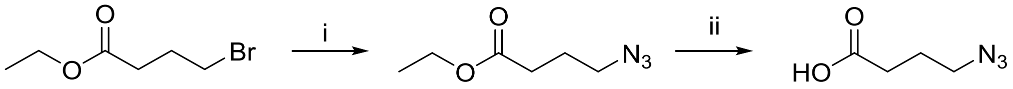

4 **5**

Reagents and conditions: i) NaN_3, DMSO, room temperature ii) NaOH (2 M), MeOH, room temperature.

Ethyl 4-azidobutyrate (4)

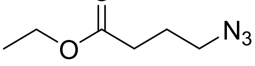

To a solution of ethyl 4-bromobutyrate (2 g, 10.3 mmol, 1 eq) in DMSO (80 mL) was added dropwise sodium azide (803 mg, 12.36 mmol, 1.2 eq) and the reaction mixture stirred overnight at room temperature. The reaction mixture was then diluted with water (20 mL) and quenched with 1 M HCl (~15 mL) dropwise. The mixture was extracted with ethyl acetate (3 x 50 mL) and the combined organic layers washed with water (3 x 50 mL) then brine (2 x 50 mL), dried over Na_2SO_4, filtered and concentrated *in vacuo*. The residue was purified by silica gel column chromatography (hexanes:Et_2O 9:1) to yield **4** as a clear oil (1.27 g, 81 % yield).^{1}H NMR (400 MHz, $CDCl_3$) δ 4.12 (q, *J* = 7.1, 2H), 3.33 (t, *J* = 6.7, 2H), 2.38 (t, *J* = 7.3, 2H), 1.89 (p, *J* = 7.0, 2H), 1.27 – 1.21 (t, *J* = 7.1, 3H); ^{13}C NMR (101 MHz, $CDCl_3$) δ 172.91, 60.78, 50.86, 31.39, 24.47, 14.41.

4-azidobutyric acid (5)

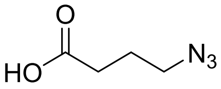

Ethyl 4-azidobutyrate (**4**) (1.2 g, 7.6 mmol, 1 eq) was suspended in 2 M NaOH (7.6 mL, 15.2 mmol, 2.0 eq) and MeOH (3 mL) was slowly added to obtain a homogeneous solution. The mixture was stirred at room temperature for 24 h, and MeOH was removed *in vacuo*. EtOAc (20 mL) was added and the aqueous layer acidified with 2 M HCl. The two layers were separated and the aqueous layer was extracted with EtOAc (2 x 50 mL). The product was extracted with Et_2O (4 x 100 mL), and the combined organic layers dried over Na_2SO_4, filtered, and concentrated *in vacuo* to give **5** as a clear oil (1.0 g, quantitative). The product did not require further purification. ^{1}H NMR (400 MHz, $CDCl_3$) δ 3.36 (t, *J* = 6.7, 2H), 2.46 (t, *J* = 7.2, 2H),

1.90 (p, *J* = 7.0, 2H); ^{13}C NMR (101 MHz, $CDCl_3$) δ 179.07, 50.66, 31.07, 24.14; HRMS (CI, positive mode) found 147.0883 ($[M + NH4]^+$ requires 147.0877).

6. Peptide Synthesis

All peptides were synthesised using an Intavis ResPrep SL Automated Peptide Synthesiser (Intavis Bioanalytical Instruments, Germany), equipped with a Mini-Column array module, utilising the Fmoc/*t*-Bu solid phase orthogonal protocol. *N*-α-Fmoc amino acid derivatives were obtained from Novabiochem (Nottingham, UK).

Resins (20 µmol) were swelled in DMF for 30 min prior to coupling. Fmoc-amino acid derivatives (100 µmol, 5 eq) pre-activated with HBTU (100 µmol, 5 eq) and NMM (200 µmol, 10 eq) in NMP were added, and incubated for 30 minutes at room temperature. The coupling mixture was removed by vacuum filtration, before the coupling reaction was repeated. The peptide was capped with a 5% v/v acetic anhydride in NMP. The resin was washed with NMP and the Fmoc group removed by addition of piperidine in NMP (20 % v/v). The resin was washed with DCM and DMF prior to the next coupling cycle.

After completion of synthesis, the resin was washed with DMF, followed by DCM, followed by methanol, followed by diethyl ether and dried overnight under vacuum in a desiccator. Peptides were cleaved from resins in TFA cleavage cocktail (1 mL) for 2 h at room temperature with agitation and the resin washed with cleavage cocktail (1 mL). The crude peptide was concentrated under a steady stream of N_2 for 15 min, and precipitated by dropwise addition to cold diethyl ether (-20 °C, 10 mL). Peptides were collected by centrifugation (4000 rpm, 5 min, 4 °C), washed with cold diethyl

ether (-20 °C, 4 x 10 mL) and dried *in vacuo* overnight. Peptides were analysed and prepared over a linear solvent gradient of 5-98% methanol (0.10% v/v formic acid) in water (0.10% v/v formic acid) for 18 min, unless otherwise stated. The fractions containing purified peptide were combined and lyophilised.

4-Azidobutyric acid-DYKDDDDK-COOH (Azido-FLAG)

Azido-FLAG peptide was synthesised on a Wang resin pre-loaded with Lysine (0.12 mmol). After the final Fmoc deprotection, 4-azidobutyric acid was manually coupled to the peptide. The resin was swelled in DMF for 1 h, and the DMF removed by filtration. 4-Azidobutyric acid (**5**) (2.5 eq) pre-activated with HATU (2.5 eq) and DIPEA (5 eq) in DMF (1 mL) was added to the resin bound peptide and shaken at room temperature for 2 h. The reagent mixture was removed by filtration and the coupling step was repeated. The resin was washed as previously described and cleaved in a TFA cleavage cocktail of (v/v) TFA 88 %, phenol 5 %, H_2O 5 %, TIPS 2 %. Az-FLAG was purified by preparative RP-HPLC as previously described. Az-FLAG-OH was obtained as a white lyophilised solid (42 mg, 31% yield). LC-MS**:** R_T=1.38 min (Absorption wavelength = 258 nm), m/z (positive mode) = 1124.51, m/z (negative mode) = 1122.65, Purity > 95 %.

CGPGRGFGKRKG-PEG$_3$-Biotin (Shh(1-11))

Shh(1-11) was synthesised on NovaTag PEG Biotin resin (20 µmol) as previously described. The peptide was cleaved in a cleavage cocktail of (v/v) 85 % TFA, 7.5 % phenol solution, 5 % H_2O, 5 % TIPS and (w/v) 2.5 % DTT. Shh was purified by

preparative RP-HPLC as previously described. Shh was obtained as a white lyophilised solid (7 mg, 21 % yield). LC-MS: R_T=6.83 min (Absorption wavelength = 258 nm), MS (ESI, m/z, positive mode): calcd. for $C_{71}H_{124}N_{24}O_{17}S_2^{2+}$ $[M+2H]^{2+}$, 824.45, found, 824.73 $[M+2H]^{2+}$, calcd. for $C_{71}H_{125}N_{24}O_{17}S_2^{3+}$ $[M+3H]^{3+}$, 549.97, found, 550.14 $[M+3H]^{3+}$, (ESI, m/z, negative mode) calcd. for $C_{71}H_{120}N_{24}O_{17}S_2^{2-}$ $[M-2H]^{2-}$, 822.44, found, 822.72 $[M-2H]^{2-}$, Purity > 92 %.

YnC$_{15}$-CGPGRGFGKRKG-PEG$_3$-Biotin (YnC$_{15}$-Shh)

Shh(1-11) was synthesised on NovaTag PEG Biotin resin (20 µmol) as previously described. After the final Fmoc deprotection, heptadec-16-ynoic acid was manually coupled to the peptide. The resin was swelled in DMF for 1 h, and the DMF removed by filtration. Heptadec-16-ynoic acid (**3**) (2.5 eq), pre-activated with HATU (2.5 eq) and DIPEA (5 eq) in DMF (1 mL) was added to the resin bound peptide and shaken at room temperature for 2 h. The reagent mixture was removed by filtration and the coupling step was repeated. The resin was washed as previously described and cleaved in a TFA cleavage cocktail of (v/v) 85 % TFA, 7.5 % phenol solution, 5 % H_2O, 5 % TIPS and (w/v) 2.5 % DTT, for 2 h at room temperature. YnC$_{15}$-Shh was purified by preparative RP-HPLC as previously described. YnC$_{15}$-Shh was obtained as a white lyophilised solid (12 mg, 42 % yield). LC-MS: R_T=11.44 min (Absorption wavelength = 258 nm), MS (ESI, m/z, positive mode): calcd. for $C_{88}H_{152}N_{24}O_{18}S_2^{2+}$ $[M+2H]^{2+}$, 948.56, found, 949.06 $[M+2H]^{2+}$, calcd. for $C_{88}H_{153}N_{24}O_{18}S_2^{3+}$ $[M+3H]^{3+}$, 632.71, found, 633.01 $[M+3H]^{3+}$, (ESI, m/z, negative mode) calcd. for $C_{88}H_{148}N_{24}O_{18}S_2^{2-}$ $[M-2H]^{2-}$, 946.54, found, 947.12 $[M-2H]^{2-}$, Purity > 90 %.

7. References

1. Wüst, F., Dence, C. S., McCarthy, T. J. & Welch, M. J. A new approach for the synthesis of [11C]-labeled fatty acids. *J. Label. Compd. Radiopharm.* **43,** 1289–1300 (2000).

2. Heal, W. P. *et al.* Bioorthogonal chemical tagging of protein cholesterylation in living cells. *Chem. Commun.* **47,** 4081–4083 (2011).
